# Assessment of Food Safety Knowledge, Attitude, Self-Reported Practices, and Microbiological Hand Hygiene of Food Handlers

**DOI:** 10.3390/ijerph14010055

**Published:** 2017-01-10

**Authors:** Hui Key Lee, Hishamuddin Abdul Halim, Kwai Lin Thong, Lay Ching Chai

**Affiliations:** 1 Institute of Biological Sciences, Faculty of Science, University of Malaya, Kuala Lumpur 50603, Malaysia; huikey90@gmail.com (H.K.L); thongkl@um.edu.my (K.L.T); 2 Occupational Safety & Health Unit, Registrar’s Department, University of Malaya, Kuala Lumpur 50603, Malaysia; hishamuddin@um.edu.my

**Keywords:** food safety, food handler, KAP, microbiological assessment, hand hygiene

## Abstract

Institutional foodborne illness outbreaks continue to hit the headlines in the country, indicating the failure of food handlers to adhere to safe practices during food preparation. Thus, this study aimed to compare the knowledge, attitude, and self-reported practices (KAP) of food safety assessment and microbiological assessment of food handlers’ hands as an indicator of hygiene practices in food premises. This study involved 85 food handlers working in a university located in Kuala Lumpur, Malaysia. The food safety KAP among food handlers (*n* = 67) was assessed using a questionnaire; while the hand swabs (*n* = 85) were tested for the total aerobic count, coliforms, and *Escherichia coli*, *Staphylococcus aureus*, *Salmonella*, *Vibrio cholerae* and *Vibrio parahaemolyticus*. The food handlers had moderate levels of food safety knowledge (61.7%) with good attitude (51.9/60) and self-reported practices (53.2/60). It is noteworthy that the good self-reported practices were not reflected in the microbiological assessment of food handlers’ hands, in which 65% of the food handlers examined had a total aerobic count ≥20 CFU/cm^2^ and *Salmonella* was detected on 48% of the food handlers’ hands. In conclusion, the suggestion of this study was that the food handlers had adequate food safety knowledge, but perceived knowledge failed to be translated into practices at work.

## 1. Introduction

The World Health Organization (WHO) reports that there approximately 2 million fatal cases of food poisoning occur every year globally [1], especially in developing countries. This scenario could be due to the poor state of food safety and general hygiene in those countries. In 2014, Malaysia recorded 49.79 cases of food poisoning per 100,000 population [2]. More than 50% of the total food poisoning cases were attributed to improper food handling by food handlers [3]. The outbreaks in academic institutions contributed 43% of the total foodborne poisoning incidents in Malaysia [4]. The Ministry of Health Malaysia [5] has identified ineffective food handling training, the use of untreated water for non-drinking purposes, and poor sanitation and hygiene as the primary risk factors of food poisoning in the country.

Food handlers play a paramount role in ensuring food safety and prevention of food poisoning. Michaels and co-workers [6] reported that infected food handlers were able to transmit agents of gastrointestinal infectious diseases via poor personal hygiene practices. A previous study successfully isolated *Salmonella* from seafood [7] but *Salmonella* is not a common carrier. This was thought to be a result of cross-contamination by infected food handlers [7]. Moreover, many reports have demonstrated similarities between the pathogens isolated from patients and food handlers, clearly indicating that food handlers were the vehicles of transmission for the foodborne pathogens [8,9]. Angelillo et al. [10] suggested that food handlers who had good knowledge of proper food handling practices could help to control food poisoning cases as they were in direct contact with food, particularly ready-to-eat foods.

Poor personal hygiene, primarily ineffective hand washing, has been recognised as a significant risk factor of food contamination that leads to food poisoning [11,12]. Hand hygiene is the most basic yet critical criterion for ensuring safe food handling by food handlers. In fact, hand washing has long been known to be a fundamental precautionary measures in health care settings [13], as well as in the kitchen, for preventing the spread of infectious disease through human to human or human to food contact [14,15,16,17]. Therefore, it is thought that hand hygiene could serve as an indicator of food handlers adherence to safe food practices during food preparation.

In Malaysia, it is mandatory under the Food Act 1983 for all food handlers to attend and complete the safe food handling course established by the Malaysian government, and they need to be vaccinated against typhoid since this disease is endemic in Malaysia. Many local studies have reported that the food handlers are mostly foreign contract workers who have adequate knowledge, positive attitudes, and good self-reported practices [18,19,20]. Nevertheless, the food poisoning rates in this country have still been increasing since 2000 [21]. The discrepancy observed between the previous research findings and epidemiological statistics have motivated us to compare the real practice of hygiene to the knowledge, attitude, and self-reported practices related to food hygiene and safety among food handlers.

Information on the food safety and hygiene practices of food handlers is scant. Thus, this study focused on assessing the food safety knowledge, attitude, and self-reported practices among food handlers via a questionnaire, in addition to hand hygiene assessment via microbiological assays. By combining both approaches, the findings provided us with a better understanding of the extent of translation of knowledge into real hygiene and safety practices as reflected in the hand hygiene of food handlers. The data generated provides an important basis for planning and determination of further approaches to be taken to improve food safety in the country.

## 2. Materials and Methods

The ethical approval was obtained from the University of Malaya Medical Ethics Committee for this study (RP003-13BIO) while informed consents were obtained from the participants.

### 2.1. Sampling Plan

This study includes all the food handlers working in canteens located in the university campus. There are 18 canteens in the university with 250–300 food handlers (inclusive of permanent and contract workers). However, we only managed to get consent from 111 food handlers to take part in the study. Of these, 41 food handlers (36.9%) participated in both the questionnaire and microbiological hand hygiene assessment; while 26 (23.4%) and 44 (39.6%) food handlers participated only in the questionnaire and microbiological hand hygiene assessment, respectively. The reason for the unbalanced sampling was because the questionnaire and hand hygiene assessment were conducted at different times and some contract workers had already left their jobs at the time that the microbiological hand hygiene assessment was conducted. Therefore, to increase the number of participants in the hand hygiene assessment, new workers who gave consent were included.

### 2.2. Questionnaire Collection

The questionnaire was in dual languages (English and Malay language) and consisted of 97 items on demographic information (11 items), food safety knowledge (60 items), attitude (14 items), and self-reported practices (12 items). Food safety knowledge was assessed based on six constructs: (1) personal hygiene; (2) cross-contamination prevention and sanitation; (3) food handling; (4) health problems that would affect food safety; (5) symptoms of foodborne diseases and (6) foodborne pathogens. Items in construct 1–4 were based on basic content taught in the safe food handling course Malaysia. The respondents were required to choose either “true” or “false” for each item on food safety knowledge and the score was given for each correct answer. The overall performance on food safety knowledge was converted to a percentage by dividing the total score over the total number of items of food safety knowledge. While the food safety attitude and self-reported practices were assessed by four-level and five-level Likert scale questions, respectively. For items under the attitude section, the lowest point (1 point) was given to “disagree” to the highest (4 points) for “agree”; while the self-reported practices were scored from the lowest (1 point) for “never” to the highest (5 points) for “always”.

We collected questionnaires from 67 food handlers of contract and/or permanent status from twelve food premises within the campus from December 2013 to August 2014. The participation of food handlers in this study was conducted on a voluntary basis. A self-administered questionnaire adapted from previous studies [22,23] was given to the literate food handlers; whereas the illiterate food handlers were assisted by a trained moderator in answering the questionnaire.

### 2.3. Hand Hygiene Assessment

#### 2.3.1. Sample Collection

A second visit to the food premises was done to collect hand swabs from the participants who had answered the questionnaire. However, during the visit, hand swabs were not performed for 26 participants who had already left the job. In the meantime, hand swabs were also collected from new workers (*n* = 44) who were willing to participate in this study. Therefore, a total of 85 hand swab samples were collected.

Each sterile swab was dipped into a falcon tube containing 10 mL of maximum recovery diluent (MRD; Merck, Darmstadt, Germany) to moisten the swab tip and then swabbed over the palm and fingertips of the food handler’s hand. A swab was collected from each food handler. The hand swabs were collected from 11.00 a.m. to 3.00 p.m. while the food handlers were serving lunch, then transported in MRD as a transport medium to the laboratory within 2 h for immediate laboratory analysis.

#### 2.3.2. Sample Enrichment and Enumeration by Plate Count Method

The hand swab was vortexed for 10 seconds to release bacteria from the cotton swab. Ten-fold dilution was made for further enumeration. Microbial loads of aerobic bacteria, coliforms, *Escherichia coli* (*E. coli*) and *Staphylococcus aureus* (*S. aureus*) were analysed using Petrifilm™ Aerobic plate count, *E. coli*/coliforms plate count, Staph Express plate count, respectively. Petrifilm™ plates were incubated as stated in the manufacturer’s instructions. For each type of microbial enumeration, a duplicate test was carried out to validate and ensure reproducibility. The average surface area of hands used in calculation of bacteria count on food handler’s hand (cfu/cm^2^) was obtained from study by Lee et al. [24], while the thresholds of the aerobic bacteria, coliforms, *E. coli*, and *S. aureus* counts were adopted from study by Tan et al. [25] and Sneed et al. [26].

#### 2.3.3. Detection and Enumeration of *Salmonella* spp., *V. cholerae* and *V. parahaemolyticus* Enumeration by Polymerase Chain Reaction (PCR)

The Most Probable Number (MPN) three-tube method was applied to quantify *Salmonella* spp., *V. cholerae* and *V. parahaemolyticus* of the hands of food handlers. Buffered peptone water (Merck) was used as the pre-enrichment broth *Salmonella* spp. whereas the alkaline peptone water (Merck) was used as an enrichment medium for *V*. *cholerae* and *parahaemolyticus.* The MRD containing bacteria from the hands of food handlers was diluted with enrichment medium and incubated overnight at 35 ± 2 °C in 1.5 mL microcentrifuge tubes. The enriched samples were subjected to DNA extraction and then Polymerase Chain Reaction (PCR) assay was used for *Salmonella* spp., *Vibrio cholerae* and *V. parahaemolyticus* detection.

DNA templates were prepared using the boiling method. A pair of primers namely OMPCF/OMPCR designed by Alvarez et al. [27], were used for *Salmonella* spp. detection while primers named pntA 1C/2C and pntA 1P/2P were used [28] for *V. cholerae* and *V. parahaemolyticus* detection. PCR products were analysed by using 1.5% of LE agarose (Promega, Madison, WI, USA) at 100 V for 30 min. All the oligonucleotides used were commercially synthesised (IDT, Coralville, IA, USA). The laboratory culture collections in this study, *Salmonella enterica* subsp. serovar Enteritidis (SE H16), *Vibrio cholerae* 86020, and *Vibrio parahaemolyticus* J 42 were used as the positive controls.

### 2.4. Statistical Analysis

Although there were 67 questionnaires and 85 hand swabs obtained, the total number of food handlers participating in both questionnaire, and microbial assessment was 41. Both questionnaire and microbial assessments were done by volunteering. Thus, there was a discrepancy in the number of samples in each part of the study. The statistical analyses were carried out using an IBM SPSS Statistics Version 22 (IBM, New York, NY, USA). Independent samples *t*-test was applied in analysing the significant differences between self-reported practices of food handling and the microbiological hygiene assessment results. The two-sided *p*-value was set at 0.05.

## 3. Results

Approximately 64.2% (*n* = 43) of the food handlers who undertook the questionnaire were aged from 21 to 41 years old; while 61.2% (*n* = 41) were of foreign nationality. More than half of the participants (*n* = 36, 53.7%) had ≥2 years’ experience in the food service industry (Table 1). Out of 67 food handlers involved in this study, a quarter (*n* = 17, 25.4%) of the food handlers had not attended the safe food handling course, which is compulsory under the Malaysian Food Act 1983; and therefore, most of these untrained food handlers were not vaccinated for typhoid fever either (Table 1).

**Figure 1:**
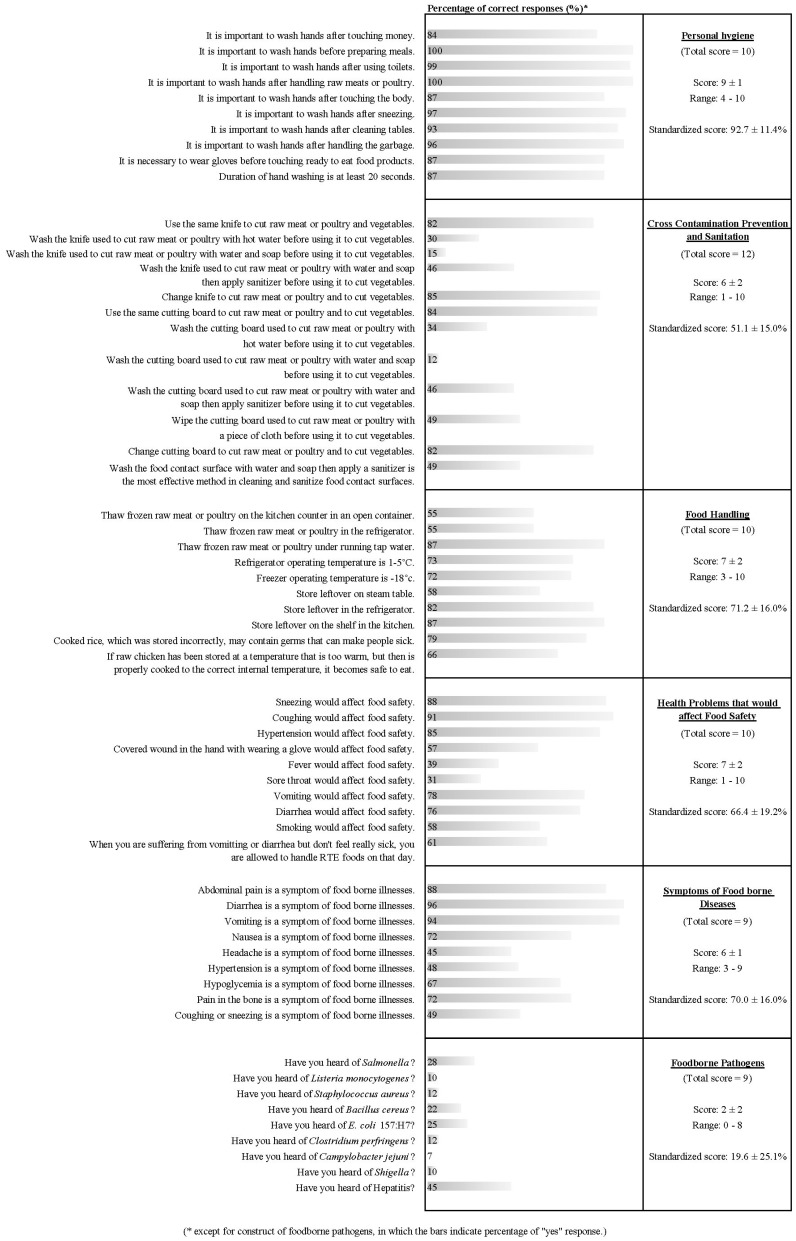
Percentage of the correct answers on food safety knowledge scored by 67 food handlers.

The food handlers demonstrated moderate overall knowledge of food safety (mean score = 61.7 ± 8.1%). Of the six constructs on food safety knowledge tested, the respondents scored highest in the construct of personal hygiene (mean score = 97.7 ± 11.4%) but performed poorly in the construct of cross-contamination prevention and sanitation (mean score = 51.1 ± 15.0%) and foodborne pathogens (mean score = 19.6 ± 25.1%; Figure 1). Based on the questionnaires, the participants showed an overall good attitude, scoring an average of 51.9 ± 4.2 out of the total score of 57 (Table 2). Also, the participants had reported that they frequently practised safe food handling during food preparation, scoring an average of 53.2 ± 5.5 of the total score of 60 (Table 3).

This study showed that the education level, working experience, and safe food handling course had different degrees of impact on food safety knowledge and attitudes of food handlers (Table 4). It is interesting to note that those who had not received any formal education performed better than those who had received primary education. Nonetheless, it was found that those who had secondary education and above scored significantly higher on food safety items related to food handling (*p* < 0.05; Table 4); and only food handlers with tertiary education knew more about foodborne pathogens than the rest (*p* < 0.05; Table 4). On the other hand, food handlers who had more working experience in the food service industry had a better overall food safety knowledge (more than 6 years > 5–6 years > 2–4 years ≥ 2 years, *p* < 0.05) than food handlers with lesser experience. From the questionnaire, even though the safe food handling course did not significantly improve the food safety knowledge, those who had attended the course performed slightly better than those who had not attended the course (Table 4). Most importantly, the safe food handling course had a significant positive impact on the attitudes toward food safety.

Hand hygiene assessment of the food handlers participating in this study revealed that 65% (*n* = 55) of them had an aerobic bacterial count exceeding the threshold of ≥20 CFU/cm^2^ based on Sneed et al. [26] and Tan et al. [24]. Approximately 35% (*n* = 30) had exceeded the standard coliform count (≥10 CFU/cm^2^; Table 5). Moreover, *Salmonella*, *V. cholerae* and *V. parahaemolyticus* were detected in 41 (48%), 2 (2%), and 1 (1%) of the 85 food handlers examined (Table 5). The number of *Salmonella* present on the contaminated hands ranged from 3 to 150 MPN/hand (Table 5).

Ironically, the food handlers who had a non-compliant coliform count (≥20 CFU/cm^2^) claimed to use gloves more frequently when touching or distributing unwrapped foods than those who had a compliance count of coliform (Table 6). On the contrary, the food handlers who were detected with the presence of *Salmonella* attested that they were less frequent in using caps while handling food (4.2 ± 1.6, *n* = 24, *p* < 0.05) than those who had negative detection for in the presence of *Salmonella*. Furthermore, the respondents who had of an exceeded limit of total aerobic bacteria (≥20 CFU/cm^2^) on their hands declared that they wash their hands before touching the unwrapped foods (4.7 ± 0.5, *n* = 27, *p* < 0.05) more frequently. The food handlers who has coliform count exceeded the threshold reported that they sanitise their working cloths more frequently (5.0 ± 0.0, *n* = 15, *p* < 0.05).

## 4. Discussion

In Malaysia, the food service industry has shown an increasing trend of hiring foreign labourers to work as servants, stewards, and cooks to prepare foods. The actual number of foreign food workers working in Malaysia is not known because most of them are working on a contract basis. The use of contract workers in food premises has raised the public concern about food operations’ ability to ensure food safety [29]. Our study reflected the scenario where there are more foreign food handlers (61.2%) working in the food premises than the locals (Table 1). Other similar studies conducted in another part of Malaysia have also reported the same scenario [19,20,29]. One of the main concerns of having more foreign food handlers in Malaysia is the effectiveness of the safe food handling course which is conducted in either Malay or English languages. The majority of the foreign food handlers are from India, Pakistan, Nepal, and Cambodia and their command of English is low and therefore it is presumed that the safe food handling course will be of little impact in instilling proper food safety practices among the food handlers. However, our findings did not reflect such a scenario. Although the improvement of knowledge performance was not significant between those who had attended the course and those who had not, the safe food handling course showed a significant impact on instilling positive food safety attitudes, particularly regarding food safety concerns among the respondents who were predominantly foreigners (Table 4). Nonetheless, more detailed work is required to review the effectiveness of the national safe food handling course, particularly regarding the foreign food workers. An easy-to-understand module such as those based on illustrations could be more efficient in delivering the knowledge to food handlers of different backgrounds and education levels.

Of the six constructs assessed on food safety knowledge, the food handlers who participated in this study demonstrated good knowledge of personal hygiene (mean score: 97.7 ± 11.4%) but not on cross-contamination (mean score: 51.1 ± 15.0%). Our finding was in agreement with other studies in Malaysia [19]. We believe that this scenario is a reflection of the current safe food handling course in Malaysia which focuses on the personal hygiene of food handlers while less emphasis is given to prevention of cross-contamination. The food handlers demonstrated poor knowledge of foodborne pathogens (19.6 ± 25.1%). This finding is supported by Liu et al. [30] Based on the study conducted by Saad and co-workers [29] on hygiene practices among food handlers in governmental institutions in Malaysia, about 30% of the food handlers commented that the safe food handling course failed to improve their knowledge at work. However, our study suggested that the food safety handling course could have significantly improved the awareness of food handlers, particularly regarding foodborne pathogens (Table 4). Nevertheless, the findings indicated that the contents of the safe food handling course need to be reviewed and improved.

The education level of food handlers is generally perceived as one of the factors that compromised the food safety and hygiene. Although we have observed an improvement in the food safety knowledge among those with tertiary education, food handlers with lower education levels, particularly those who had no formal education outperformed those with higher education (Table 4). For instance, the food handlers without formal education outperformed others on personal hygiene knowledge (Table 4). These findings are further supported by the Pichler et al. [23], McIntyre et al. [31], Lynch et al. [32] and Toh and Birchenough [33]. Working experience, on the other hand, was found to have a significant impact on the overall food safety knowledge among the respondents in this study (Table 4). Saad and co-workers [29] also reported a similar observation.

In this study, a microbial assessment to examine the hand hygiene of the participating food handlers was conducted to obtain a better insight into the current food safety practices in food premises. The microbiological hygiene assessment reflects the real practices of proper safe food handling and at the same time could be used to validate the self-reported practices. The findings from our study were not encouraging as many food handlers were found to have microbial counts exceeding the standards (Table 5). More alarmingly, *Salmonella* was detected on the hands of about half of the participated food handlers. These food handlers could be the asymptomatic carriers for *Salmonella* transmission as *Salmonella* can remain in a carrier state up to 300 days after infection [34]. This raises many public health concerns, as most of the food handlers were not wearing gloves during food handling, as we observed during the study. This situation could eventually increase the risk of food poisoning.

Other studies have reported that provision of food safety and hygiene knowledge is not necessarily translated into safe food behaviour or practice [10,35,36,37]. Similarly, our results indicated that the generally moderate performance on food safety knowledge was not reflected in the microbial hand hygiene assessment. For instance, the respondents who claimed that they often wore gloves had coliform counts exceeding the threshold (Table 6). Our observation did not suggest that the food handlers often wore gloves. This scenario could eventually increase the risk of coliform contamination in foods. Overall, the findings suggested that the safe food handling course did, in fact, impart some knowledge and awareness of food safety, but failed to change the safe food behaviour among the food handlers. According to Worsfold et al. [38], behaviour change in safe food handling can be attained when the knowledge and skills learned are being rehearsed and used. Continual training and management support are important elements in the transfer of knowledge into behaviour [39]. In this case, further studies are required to understand the factors that have limited the transfer of knowledge into safe food practice among food handlers.

## 5. Conclusions

Although the food handlers had a moderate level of food safety knowledge with a good attitude, and self-reported practices, the poor performance in the hand hygiene assessment indicated a failure in actually practising safe food handling in their job. The contaminated hands of food handlers could easily transmit foodborne diseases through cross-contamination of food products. The current findings indicated a need to review the effectiveness of the current national safe food handling course, not just to improve the effectiveness to disseminate food safety knowledge and attitudes, but also to induce a real change in safe food handling behavior among food handlers by taking into consideration the multi-cultural and education level of food handlers in Malaysia. It is apparent from this study that a good knowledge in food safety is not indicative of food safety practice in the real world. Studies are required to look into the factors that inhibit the transfer of knowledge into food safety behaviour in Malaysia so that an effective food safety policy can be put in place.

There are several limitations in this research. These results only relied on the very limited number of food handlers who took part in this study and therefore the observation or findings in the study should not be extrapolated to represent the scenario in Malaysia. However, this study is exploratory in nature and serves as a pilot study for a nationwide study of the relationship between hand sanitation and the KAP of food handlers. Future studies should involve a collaboration with the government and a larger population of food handlers so that the authority can establish a more comprehensive approach to ensure food safety.

## Figures and Tables

**Table 1:** Participant demographic characteristics.

Variable	Item	Number	Percentage (%)
Gender	Male	27	40.3
	Female	40	59.7
Age	<21 years old	5	7.5
	21–41 years old	43	64.2
	>41 years old	19	28.4
Nationality	Malaysian	26	38.8
	Foreigner	41	61.2
Marital Status	Single	14	20.9
	Married	49	73.1
	Divorce	4	6.0
Education level	No formal education	11	16.4
	Primary school	7	10.5
	Secondary school	37	55.2
	College/University	12	17.9
Work experience	<2 years	31	46.3
	2–4 years	16	23.9
	5–6 years	9	13.4
	>6 years	11	16.4
Job responsibility	Cooking	35	52.3
	Cleaning and washing dishes	3	4.5
	Serving food	17	25.4
	Preparation of food ingredients	4	6.0
	Others (cashier, manager, etc.)	8	11.9
Did you attend the Safe Food Handling course?	No	17	25.4
	Yes	50	74.6
When did you attend the Safe Food Handling course?	Never attend before	17	25.4
	≤3 years ago	29	43.3
	>3 years ago	21	31.3
Total		67	100

**Table 2:** Participant food safety attitude scores.

Item		Mean	SD	Min	Max
Self-improvement					
	I would read more journals about food safety in order to enhance my food sanitation knowledge.	3.5	0.9	1.0	4.0
	I think by attending a sanitation seminar, it would increase my sanitation knowledge and ideas.	3.9	0.4	1.0	4.0
	I would attend a cooking or service competition to improve my professional knowledge.	3.2	1.2	1.0	4.0
	I would attend food safety seminar to gain more food safety knowledge.	3.8	0.5	1.0	4.0
	I think I do not need to attend food safety seminar because I think I have sufficient knowledge about food safety.	1.6	1.6	1.0	4.0
Food safety concern					
	Food handlers are responsible to prevent food poisoning.	3.9	0.5	1.0	4.0
	Government is responsible to prevent food poisoning.	3.2	1.3	1.0	4.0
	University is responsible to prevent food poisoning.	3.3	1.2	1.0	4.0
	Consumers are responsible to prevent food poisoning.	3.4	1.1	1.0	4.0
	Maintaining a clean cooking environment is a good way to control food safety.	4.0	0.3	2.0	4.0
	Self-checking of food safety is important to restaurants and institutions.	3.8	0.4	2.0	4.0
	Food safety is more important than taste.	3.8	0.4	2.0	4.0
	Food safety knowledge is important to ensure food is prepared in a safe manner.	3.9	0.5	1.0	4.0
	Food poisoning is not a serious matter.	1.7	1.3	1.0	4.0

**Table 3:** Participant self-reported food safety practices.

Item	Mean	SD	Min	Max
Do you wash your hands before touching unwrapped raw foods?	4.6	0.8	1.0	5.0
Do you wash your hands after touching unwrapped raw foods?	4.6	0.9	1.0	5.0
Do you use gloves when you touch or distribute unwrapped foods?	4.3	1.2	1.0	5.0
Do you use protective clothing (apron) when you touch or distribute unwrapped foods?	4.6	1.0	1.0	5.0
Do you use mask when you touch or distribute unwrapped foods?	2.8	1.5	1.0	5.0
Do you use cap when you touch or distribute unwrapped foods?	4.5	1.2	1.0	5.0
Do you use different chopping board for raw meat and fresh produce (vegetables and fruit)?	4.3	1.1	1.0	5.0
Do you wash and sanitise the working clothes?	4.8	0.6	1.0	5.0
Do you use a different cloth or towel to dry plates?	4.6	1.0	1.0	5.0
Do you wash and sanitise the knife after chopping raw chicken or meat?	4.8	0.4	4.0	5.0
Do you use clean and washed plate for ready-to-eat foods?	4.9	0.3	4.0	5.0
Do you work when you are sick (flu, cold, diarrhoea, coughing, etc.)?	4.3	1.1	1.0	5.0

**Table 4:** Attribution of food safety knowledge, attitude, and self-reported scores to educational level, work experience, and safe food handling course of participants (*n* = 67).

Construct	Education Level				Work Experience				Did You Attend the Safe Food Handling Course?		When Did You Attend the Safe Food Handling Course?		
	No Formal Education	Primary School	Secondary School	College/University	≤2 Years	2–4 Years	5–6 Years	>6 Years	No	Yes	Never Attended	≤3 Years Ago	>3 Years Ago
	(*n* = 11)	(*n* = 7)	(*n* = 37)	(*n* = 12)	(*n* = 31)	(*n* = 16)	(*n* = 9)	(*n* = 11)	(*n* = 17)	(*n* = 50)	(*n* = 17)	(*n* = 29)	(*n* = 21)
Knowledge (%)													
Personal hygiene	95.5 ^a^	87.1 ^a^	94.3 ^a^	88.3 ^a^	92.3 ^a^	91.3 ^a^	94.4 ^a^	94.6 ^a^	90.0 ^a^	93.6 ^a^	90.0 ^a^	94.8 ^a^	91.9 ^a^
Cross contamination prevention and sanitation	49.2 ^a^	60.7 ^a^	49.8 ^a^	53.5 ^a^	50.5 ^a^	53.1 ^a^	50.9 ^a^	52.3 ^a^	48.0 ^a^	52.7 ^a^	48.0 ^a^	53.5 ^a^	51.6 ^a^
Food handling	**66.4** ^a,b^	**52.9** ^a^	**75.1** ^b^	**74.2** ^b,c^	**73.2** ^a,b^	**61.3** ^a^	**66.7** ^a,b^	**83.6** ^b^	73.5 ^a^	70.4 ^a^	73.5 ^a^	68.3 ^a^	73.3 ^a^
Health problems that would affect food safety	62.7 ^a^	54.3 ^a^	69.2 ^a^	68.3 ^a^	61.9 ^a^	63.1 ^a^	73.3 ^a^	78.2 ^a^	72.9 ^a^	64.2 ^a^	72.9 ^a^	60.0 ^a^	70.0 ^a^
Symptoms of foodborne diseases	78.8 ^a^	66.7 ^a^	66.1 ^a^	75.9 ^a^	66.7 ^a^	72.2 ^a^	70.4 ^a^	75.8 ^a^	66.0 ^a^	71.3 ^a^	66.0 ^a^	68.6 ^a^	75.1 ^a^
Foodborne pathogens	**6.1** ^a^	**25.4** ^a,b^	**16.2** ^a^	**38.9** ^b^	14.7 ^a^	19.4 ^a^	19.8 ^a^	33.3 ^a^	**4.6** ^a^	**24.7** ^b^	**4.6** ^a^	**23.8** ^b^	**25.9** ^b^
Overall knowledge score	60.0 ^a^	58.3 ^a^	62.1 ^a^	66.4 ^a^	**60.2** ^a^	**60.3** ^a^	**62.8** ^a,b^	**69.5** ^b^	59.6 ^a^	63.0 ^a^	59.6 ^a^	61.7 ^a^	64.7 ^a^
Food safety attitude													
Self-improvement	**3.5** ^a,b^	**3.5** ^a,b^	**3.7** ^a^	**3.3** ^b^	3.6 ^a^	3.5 ^a^	3.5 ^a^	3.9 ^a^	3.6 ^a^	3.6 ^a^	3.6 ^a^	3.6 ^a^	3.5 ^a^
Food safety concern	3.5 ^a^	3.6 ^a^	3.4 ^a^	3.5 ^a^	3.4 ^a^	3.5 ^a^	3.5 ^a^	3.4 ^a^	**3.1** ^a^	**3.5** ^b^	**3.1** ^a^	**3.5** ^b^	**3.5** ^b^
Overall attitude score	3.5 ^a^	3.6 ^a^	3.5 ^a^	3.4 ^a^	3.4 ^a^	3.5 ^a^	3.5 ^a^	3.6 ^a^	**3.3** ^a^	**3.5** ^b^	**3.3** ^a^	**3.6** ^b^	**3.5** ^a,b^
Self-reported practices													
Overall practices score	4.3 ^a^	4.2 ^a^	4.2 ^a^	4.0 ^a^	4.3 ^a^	4.2 ^a^	4.0 ^a^	4.3 ^a^	4.3 ^a^	4.2 ^a^	4.3 ^a^	4.2 ^a^	4.1 ^a^

The values expressed in the column for the knowledge section are the mean percentage of each category in respect to each construct of KAP while the values expressed in the food safety attitudes and self-reported practices are the mean score obtained from the questionnaire analysis. Values in the same row and subtable not sharing the same superscript (^a^, ^b^, ^c^) are significantly different at *p* < 0.05 in the two-sided test of equality for column means. Cells with no superscript are not included in the test. Tests assume equal variances and are adjusted for all pairwise comparisons within a row of each innermost subtable using the Bonferroni correction.

**Table 5:** Performance of participants’ hand hygiene based on aerobic bacteria, coliforms and *E*. *coli*, *S*. *aureus* count and the detection of *Salmonella*, *V. cholerae* and *V. parahaemolyticus*.

**Microbial Indicator**	**Status**	**Number**	**Percentage (%)**	**Min ^a^ (CFU/cm^2^)**	**Max ^a^ (CFU/cm^2^)**
Aerobic count ^b^					
	>Threshold	55	65	23	>29
	<Threshold	30	35	<1	19
Coliform ^c^					
	>Threshold	30	35	20	>29
	<Threshold	55	65	<1	17
*E. coli* ^d^					
	>Threshold	2	2	12	13
	<Threshold	83	98	n.d.	8
*S. aureus* ^e^					
	>Threshold	3	4	11	>29
	<Threshold	82	96	n.d.	5
**Foodborne Pathogens**	**Presence**	**Number**	**Percentage (%)**	**MPN_min_ per Person ^f^**	**MPN_max_ per Person ^f^**
*Salmonella*					
	Detected	41	48	3	150
	Not detected	44	52	<3	n.a.
*V. cholerae*					
	Detected	2	2	3	n.a.
	Not detected	83	98	<3	n.a.
*V. parahaemolyticus*					
	Detected	1	1	23	n.a.
	Not detected	84	99	<3	n.a.

n.d.: Not detected or below detection limit; n.a.: Not applicable; MPN: Most probable number; ^a^ The minimum (Min) and maximum (Max) CFU/ cm^2^; ^b^ Aerobic count threshold based on Tan et al. [25] and Sneed et al. [26], which is ≥20 CFU/cm^2^; ^c^ Coliform count threshold based on Tan et al. [25] and Sneed et al. [26], which is ≥20 CFU/cm^2^; ^d^ *E. coli* count threshold based on Tan et al. [25] and Sneed et al. [26], which is ≥10 CFU/cm^2^; ^e^ *S. aureus* count threshold based on Tan et al. [25] and Sneed et al. [26], which is ≥10 CFU/cm^2^; ^f^ The minimum (MPN_min_)and maximum (MPN_max_) MPN value per person.

**Table 6:** Self-reported practices vs. microbial contamination on food handlers’ hands.

Item	Aerobic Count ^a^		Coliforms ^a^		*E. coli* ^b^	*S. aureus* ^a^		*Salmonella*	
	>Threshold (*n* = 27)	<Threshold (*n* = 14)	>Threshold (*n* = 15)	<Threshold (*n* = 26)	<Threshold (*n* = 41)	>Threshold (*n* = 2)	<Threshold (*n* = 39)	>Threshold (*n* = 24)	<Threshold (*n* = 17)
Do you wash your hands before touching unwrapped raw foods?	**4.7 ± 0.5**	**4.1 ± 0.9**	4.7 ± 0.5	4.4 ± 0.9	4.5 ± 0.7	5.0 ± 0.0	4.5 ± 0.8	4.7 ± 0.6	4.4 ± 0.8
Do you wash your hands after touching unwrapped raw foods?	4.6 ± 1.0	4.6 ± 0.9	4.4 ± 1.2	4.6 ± 0.8	4.6 ± 1.0	5.0 ± 0.0	4.5 ± 1.0	4.4 ± 1.2	4.6 ± 0.8
Do you use gloves when you touch or distribute unwrapped foods?	4.3 ± 1.2	3.9 ± 1.2	**4.7 ± 0.7**	**3.9 ± 1.4**	4.2 ± 1.2	5.0 ± 0.0	4.2 ± 1.2	4.2 ± 1.4	4.2 ± 1.1
Do you use protective clothing (apron) when you touch or distribute unwrapped foods?	4.4 ± 1.2	4.6 ± 0.7	4.1 ± 1.5	4.7 ± 0.7	4.5 ± 1.1	5.0 ± 0.0	4.5 ± 1.1	4.3 ± 1.2	4.6 ± 1.0
Do you use mask when you touch or distribute unwrapped foods?	2.6 ± 1.4	2.5 ± 1.2	3.1 ± 1.5	2.3 ± 1.1	2.6 ± 1.3	3.5 ± 0.7	2.5 ± 1.3	2.6 ± 1.4	2.8 ± 1.3
Do you use cap when you touch or distribute unwrapped foods?	4.7 ± 1.1	4.6 ± 1.1	4.7 ± 1.0	4.7 ± 1.1	4.7 ± 1.1	5.0 ± 0.0	4.6 ± 1.1	**4.2 ± 1.6**	**4.9 ± 0.3**
Do you use different chopping board for raw meat and fresh produce (vegetables and fruit)?	4.3 ± 1.2	4.0 ± 0.9	4.4 ± 1.2	4.1 ± 1.1	4.2 ± 1.1	5.0 ± 0.0	4.2 ± 1.1	4.5 ± 0.8	4.2 ± 1.1
Do you wash and sanitise the working clothes?	4.9 ± 0.3	4.4 ± 1.1	**5.0 ± 0.0**	**4.6 ± 0.9**	4.7 ± 0.7	5.0 ± 0.0	4.7 ± 0.7	4.8 ± 0.4	4.6 ± 0.9
Do you use a different cloth or towel to dry plates?	4.5 ± 1.2	4.3 ± 1.1	4.7 ± 1.0	4.3 ± 1.2	4.4 ± 1.2	5.0 ± 0.0	4.4 ± 1.2	4.6 ± 0.9	4.5 ± 1.1
Do you wash and sanitise the knife after chopping raw chicken or meat?	4.9 ± 0.3	4.4 ± 0.5	**4.9 ± 0.3**	**4.7 ± 0.5**	4.8 ± 0.4	5.0 ± 0.0	4.8 ± 0.4	4.8 ± 0.4	4.7 ± 0.5
Do you use clean and washed plate for RTE foods?	4.9 ± 0.3	4.9 ± 0.3	4.9 ± 0.3	4.9 ± 0.3	4.9 ± 0.3	5.0 ± 0.0	4.9 ± 0.3	4.9 ± 0.3	4.9 ± 0.3
Do you work when you are sick (flu, cold, diarrhoea, coughing, etc.)?	4.3 ± 1.2	3.6 ± 1.1	**4.5 ± 0.7**	**3.8 ± 1.3**	4.0 ± 1.2	3.0 ± 2.8	4.1 ± 1.1	4.2 ± 1.3	4.0 ± 1.1

The values are the mean score obtained from questionnaire analysis. Results are based on two-sided tests with significance level at 0.05. The significant pair is highlighted in bold. ^a^ Threshold of aerobic bacteria count, coliforms, *E. coli* and *S. aureus* were based on Tan et al. [25] and Sneed et al. [26], which is ≥ 20 CFU/cm^2^; ^b^ *t*-test was not conducted as the one of the sample size is too little for confident statistical analysis.
